# Subsurface calcification of hydrophilic refractive multifocal intraocular lenses with a hydrophobic surface: A case series

**DOI:** 10.1097/MD.0000000000018379

**Published:** 2019-12-16

**Authors:** Seung Pil Bang, Kun Moon, Jong-Ho Lee, Jong Hwa Jun, Choun-Ki Joo

**Affiliations:** a Department of Ophthalmology, Keimyung University School of Medicine, Daegu; b Department of Biomedical Engineering, University of Rochester, Rochester, NY, United States; c Balgeunsesang Eye Clinic, Seoul; d Department of Ophthalmology and Visual Science, Seoul St. Mary's Hospital, College of Medicine, the Catholic University of Korea; e Catholic Institute for Visual Science, Catholic University of Korea, College of Medicine, Seoul, Korea.

**Keywords:** calcification, hydrophilic, multifocal, opacification, subsurface

## Abstract

**Rationale:**

Opacification of monofocal intraocular lenses (IOLs) of various designs and materials has been reported. Hydrophilic acrylic IOLs are more prone to opacification than hydrophobic IOLs, but IOL surface modification by hydrophobic materials may improve biocompatibility, and few opacifications of such monofocal lenses have been reported to date. However, here we describe the characteristics of opacification of hydrophilic refractive multifocal IOLs with a hydrophobic surface modification in a cluster of patients who underwent uneventful cataract surgery.

**Patient Concerns:**

In this retrospective observational case series, the medical records of 7 patients in whom opacification of the IOL was identified after implantation of LS-313 MF30 (Lentis M plus, Oculentis), from November 2017 to May 2019, were reviewed.

**Diagnosis:**

All patients had undergone bilateral implantation of LS-313 MF30 IOLs. Ten eyes of 7 patients showed significant opacification at a mean 49.1 ± 10.2 months postoperatively.

**Interventions:**

The IOLs of 4 cases were explanted.

**Outcomes:**

All of the opacified cases had received LS-313 MF30 IOLs from February 2014 to August 2014 and experienced decreased visual acuity after 44.6 ± 10.5 months. The explanted IOLs of 4 cases were evaluated by scanning electron microscopy (SEM), energy-dispersive X-ray spectroscopy (EDX), Alizarin-red, and von Kossa staining. These explanted IOLs showed fine and evenly distributed, whitish deposits on the entire IOL, particularly below the surface. Although the constituent of the deposits was identified as calcium by Alizarin-red and von Kossa stain, SEM, and EDX analysis showed no surface deposits of calcium. Paraffin-embedded sections of the IOLs were prepared, and calcium deposition was confirmed by EDX analysis at the subsurface region of the IOL.

**Lessens:**

Significant opacification of these hydrophilic refractive multifocal IOLs with hydrophobic surface modification was found to be due to abnormal calcification of the subsurface of the IOL. Clinicians must be aware of the opacification of this IOL design, despite surface modification. In particular, it should be noted that there is a high likelihood that the patient may experience vision-related symptoms even with moderate opacity and that opacification may lead to a burdensome IOL exchange.

## 1 Introduction

Opacification of hydrophilic intraocular lenses (IOLs) of various designs has been reported during the past 2 decades.^[1–4]^ The etiological factors of IOL opacification can be divided into primary or secondary causes. Primary opacification of hydrophilic IOLs has been reported for 4 major bands: Hydroview (Bausch & Lomb; Rochester, NY), MemoryLens (Ciba Vision; Duluth, GA), SC60B-OUV (Medical Developmental Research; Clearwater, FL), and Aqua-Sense (Ophthalmic Innovations International, Claremont, CA). In these cases, packaging, polishing, and materials were identified as causative factors.^[5–12]^ On the other hand, most cases of secondary opacification have been related to intracameral gas (sulfur hexafluoride or perfluoropropane) or air that is injected during Descemet's stripping endothelial keratoplasty, Descemet's membrane endothelial keratoplasty, or the treatment procedure for Descemet's membrane detachment after cataract surgery.^[1–4]^ In these cases, the optic surface exposed through the pupillary aperture showed focal opacification, and it is suspected that this is due to local damage to the IOL optic surface by direct contact with air or gas.^[13]^

Recently, implantation of multifocal IOLs during cataract surgery has become popular with ophthalmic surgeons and patients. Although various complications have been associated with the use of these multifocal IOLs, there have been few reports on opacification of multifocal IOLs. Nevertheless, recently, abrupt and transient clouding of hydrophilic multifocal IOLs and solitary cases of opacified hydrophobic multifocal IOLs have been reported^[14,15]^; however, late postoperative opacification of multifocal IOLs has not yet been reported.

The Lentis M plus MF30 refractive multifocal IOL is a hydrophilic acrylic IOL with hydrophobic surface modification. This IOL allows seamless transitions between the near and far vision zones. Unlike the Lentis monofocal IOL LS-312 with a c-loop haptic design, the Lentis M plus MF30 lens has a plate haptic design that facilitates centering, rotational stability, and better refractive predictability of the IOL, but uses the same optic design and material as the LS-312.^[16]^ In late 2017, the Lentis M plus MF30 lenses were subject to a voluntary recall by the manufacturer due to the possibility of calcification of the lenses produced during a certain period. In that notice, the manufacturer suggested that a phosphate-containing agent used for IOL cleansing could induce calcific deposits. Nevertheless, no opacification of these multifocal IOLs implanted in that period has been reported in the literature to date.

Here, we report a cluster of cases with opacifications of hydrophilic acrylic refractive multifocal IOLs with a hydrophobic surface modification (LS-313 MF30 IOLs).

## 2 Patients and methods

This retrospective case-series study was performed at Dongsan Medical Center, School of Medicine, Keimyung University, Daegu, South Korea. We retrospectively collected clinical data from 10 eyes of 7 patients, from November 2017 to May 2019. These patients were referred from local clinics due to clinically significant opacified IOLs in unilateral or bilateral eyes and visual quality deterioration, such as the development of glare and halo, after uneventful bilateral cataract surgery with in-the-bag implantation of hydrophilic acrylic refractive multifocal IOLs with a hydrophobic surface (Lentis M plus, LS-313 MF30, Oculentis GmbH, Berlin, Germany).

We obtained approval from the institutional review board of Dongsan Medical Center, Keimyung University (Approval no. 2019-05-062), and the patients provided informed consent for publication of the case series. From the medical records, patient data on best corrected visual acuity, concomitant medical or ophthalmic conditions, and medication, symptom duration, date of cataract surgery, IOL serial number, intraoperative complications, and additional procedures were collected. Results of assessments using the optical quality analysis system (OQAS, Visiometrics SL, Terrassa, Spain) and anterior segment optical coherence tomography (AS-OCT, DRI Triton, Topcon, Tokyo, Japan) were also collected.

The 10 eyes of the 7 patients showed significant opacification on slit-lamp examination; 4 opacified IOLs (right eye of case 4 and the left eye of cases 2, 3, and 6) were explanted and new 3-piece hydrophobic IOLs were reimplanted in-the-bag or sulcus. During the IOL exchange, ophthalmic viscoelastic devices were injected into the anterior chamber and the anterior capsular margin was carefully dissected from both the IOL optic and haptic using an iris spatula. Since the Lentis M plus LS-313 MF30 IOL has a relatively thick plate design and the total volume of the IOL is larger than that of other c-loop IOLs, a longitudinal incision was made using microforceps and Vannas scissors. Through a 2.85-mm clear corneal incision, opacified IOL was removed and the new IOL implanted in-the-bag (case 3). In posterior capsulotomized cases (cases 2, 4, and 6), 3-piece hydrophobic IOLs (Sensar 3-piece, AR40e, Johnson & Johnson Vision, Santa Ana, CA) were implanted in the ciliary sulcus after the removal of the prolapsed vitreous fibers by pars plana vitrectomy.

Explanted IOLs were photographed using a stereoscopic microscope (SZ61TR, Olympus Corporation, Shinjuku, Tokyo, Japan) and imaging software (iSolution Lite image analyzer, IMT i-solution Inc., Burnaby, BC, Canada). In addition, the explanted IOLs were evaluated by scanning electron microscope (SEM) and energy-dispersive X-ray spectroscopy (EDX) at the Korea Basic Science Institute of Daegu, to assess the deposited materials. EDX analysis was performed on both the surface and on sections of IOLs.

Explanted IOLs were stained with 2% Alizarin-red and 5% silver nitrate solution. For these procedures, explanted IOLs were rinsed 3 times with distilled water and stained with 2% Alizarin red solution for 15 minutes or 5% silver nitrate solution for 2 hours. Lenses were again washed 3 times with distilled water, and the stained IOLs were observed under the microscope. In addition, to identify the subsurface depositions, the paraffin-embedded sections of the IOLs were prepared subjected to EDX and calcium staining. To this end, a piece of IOL optic was embedded in melted paraffin, and sliced into 10-μm thick sections. EDX analysis and 2% Alizarin-red or 5% silver nitrate staining of the sections were then performed with the section. Quantitative variables were summarized as the mean and standard deviation.

## 3 Results

The mean age of patients at initial surgery was 54.1 ± 7.1 (range: 44–67) years and the mean age of the patients at diagnosis of opacification of the implanted IOL was 58.0 ± 7.3 (range: 47–71) years. The mean time from IOL implantation to opacification was 49.1 ± 10.2 months. Mean symptom duration was 2.7 ± 1.8 (range: 1–6) months. There were 3 female and 4 male patients. Two female and 2 male patients underwent IOL exchange surgeries.

**Figure 1:**
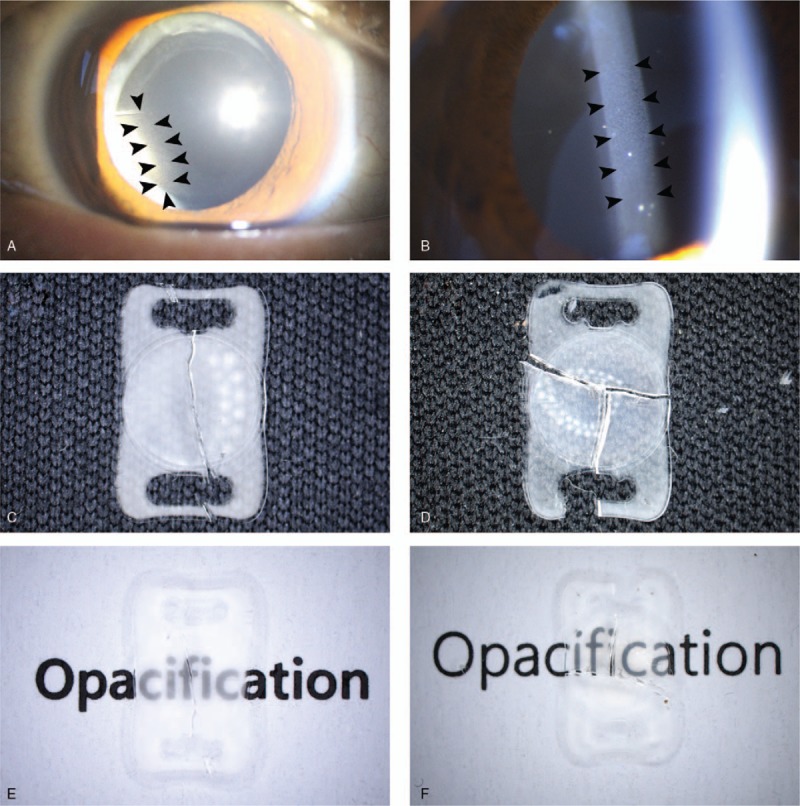
Photographs of opacified intraocular lens (IOL). A and B, Slit-lamp photographs of opacified IOL of cases 1 and 3, respectively. Fine whitish granular calcium deposits are present over the entire optic of the IOL (arrowheads). C and D, Microscopic photographs of explanted IOLs in cases 4 and 6, respectively. Calcium deposits evenly involved both the optic and haptic areas. E and F, Evaluation of disturbance in optical quality using text. Significant opacification decreased visibility of letters.

On slit-lamp examination, opacification evenly involved the entire visible optic area of all affected IOLs (Fig. 1A and B). Upon microscopic examination after explantation surgery, these calcific deposits were also seen to be distributed evenly on all IOL subsurfaces, with no un-opacified areas. The calcified IOL optics showed significantly reduced clarity (Fig. 1C–F).

Cases 1, 3, and 4 had no associated medical and ophthalmic history except dry eye disease and were taking no medicines. Cases 6 and 7 had type 2 diabetes and high myopia at initial cataract surgery (case 6: −14.0 diopter [D] in both eyes, case 7: −6.0 D in right eye, and −9.25 D in left eye, by spherical equivalent). Case 6 demonstrated poor glucose control at preoperative medical evaluation and a random blood glucose level was 297 mg/dL. In cases with bilateral IOL opacifications, the degree of opacification and symptom recognition differed between the eyes of each patient, although the less-affected eye also showed observable opacification.

**Figure 2:**
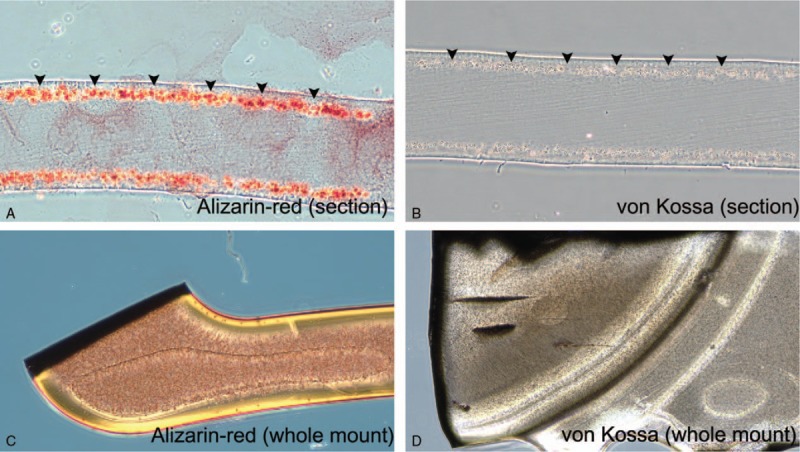
Identification of calcium depositions of intraocular lenses (IOLs). A and B, Fine granular calcium deposits were identified under the surface of IOL by both staining methods (arrowheads). C and D, Calcium deposits evenly involved the entire IOL, except for the surface of the IOL. (A and C, Two percent alizarin-red staining of IOL. B and C, Five percent silver nitrate staining of von Kossa.).

**Figure 3:**
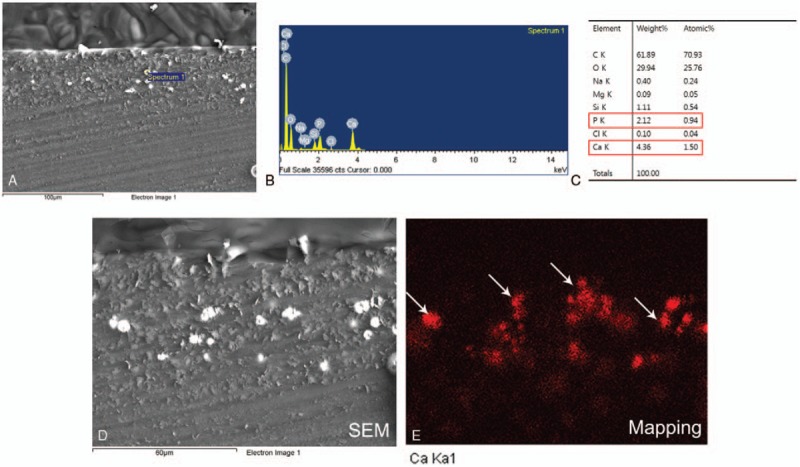
Scanning electron microscopy (SEM) and energy-dispersive X-ray spectroscopy (EDX) were performed on paraffin-embedded sections of the intraocular lens. A, Several calcific deposits were identified by SEM. B and C, EDX analysis confirmed deposits as containing calcium. D and E, Ca Ka1 shows mapping of the calcium deposits (arrows).

SEM evaluation of the IOL surfaces identified no deposit on the surface of all of the explanted IOLs, and EDX analysis identified no calcium-dominant ingredient from these IOL surfaces. However, on microscopic evaluation, Alizarin-red and von Kossa staining showed fine calcific deposits, in both the optic and haptic areas of the anterior and posterior subsurfaces of these IOLs (Fig. 2). We performed repeated EDX analysis on serial IOL sections, and confirmed the deposition of calcium in the anterior and posterior subsurfaces of the IOLs (Fig. 3).

**Figure 4:**
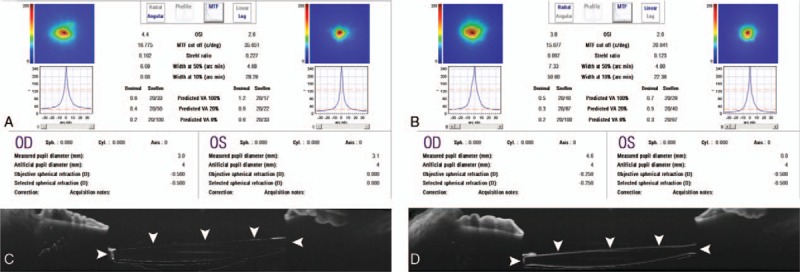
Clinical evaluations of the effects of calcification of intraocular lenses (IOLs). A and B, The results of optical quality analysis system (OQAS) of both eyes in cases 1 and 7. The IOL of the right eyes of cases 1 and 7 showed denser opacification than that of the left eye at initial examination. OQAS showed a decreased modulation-transfer function cut-off value and Strehl ratio in the right eye than in the lesser-affected left eye of both cases. The objective scattering index was also higher in the right eye of both cases. C and D, Anterior segment optical coherence tomography (AS-OCT) of the IOL in case 3. AS-OCT imaging showed higher reflectivity of the IOL surface of the left eye than of the right eye (arrowheads).

In the preoperative ophthalmic evaluation, OQAS evaluation of cases 1 and 7 showed lower modulation-transfer function (MTF) cut-off values and Strehl ratios in the right eye with the opacified IOL, but the objective scattering index was higher than in the less-affected left eye. Preoperative AS-OCT of case 3 showed significant hyper-reflectivity of the opacified IOL surface in left eye (Fig. 4).

Although all 7 patients showed significant visual deterioration in 1 eye at the initial visit, only 4 patients desired IOL exchange surgery due to symptom deterioration, and 1 patient was lost during the follow-up period. After the IOL exchange surgery, none of these 4 patients reported any visual symptoms. The clinical presentations of the 7 patients are summarized in Tables 1 and 2.

**Table 1:**
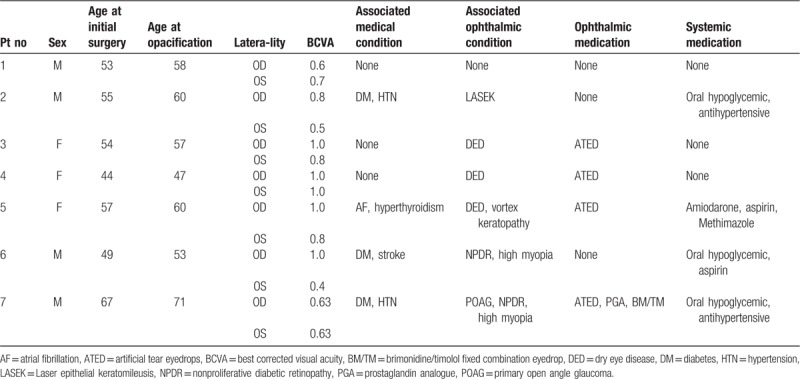
The clinical characteristics of 14 eyes of 7 patients that were referred due to IOL opacification.

**Table 2:**
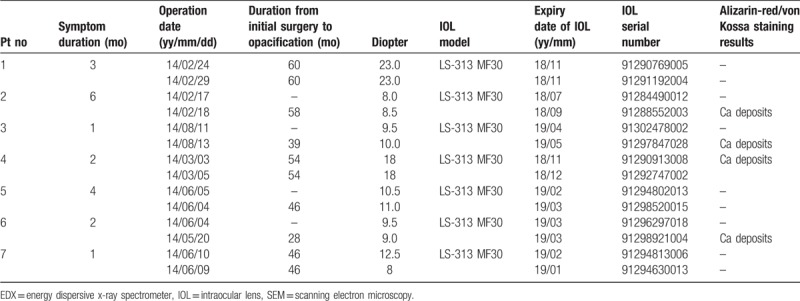
The details of parameters related to IOL implantation in 14 eyes of 7 patients that were referred due to IOL opacification.

## 4 Discussion

Hydrophilic acrylic IOLs have high water content levels to ensure sufficient flexibility to allow their insertion via a small incision. According to the literature reported to date, IOL opacification has mainly been limited to hydrophilic IOLs,^[17,18]^ with multifactorial causes.^[5,8,9,19]^ Although the exact mechanism remains unknown, the hydroxyl groups present in the poly-acrylic substance of the IOL surface could be ionized at the physiological pH of the aqueous humor, which could accelerate precipitation of calcium phosphate crystals on the IOL surface.^[20]^ To avoid this, a new type of IOL was developed in which a hydrophilic acrylic body was combined with a hydrophobic surface.

Nevertheless, clustered cases of IOL opacification in hydrophilic acrylic monofocal IOLs with a hydrophobic surface have recently been reported for IOLs manufactured from the same material.^[20–22]^ These reports described clustered cases of 1-piece or 3-piece, plate-haptic or c-loop haptic, monofocal IOLs (Oculentis GmbH, Berlin, Germany), which were made of the same material, and using the same production process, as the multifocal IOLs in our cases. Given that our clustered cases were associated with expensive multifocal IOLs, however, it was considerably difficult to persuade patients to undergo an IOL exchange after explantation, given the cost of the procedure and the risk of inserting a new multifocal IOL.

There are some distinctive features of the cases in the present report. First, precipitates were found throughout the entire optic area, including the posterior surface of the IOL, rather than only at the exposed optic portion, in all patients, including the remaining 3 unexplanted cases, based on slit-lamp microscopy. Furthermore, the opacification was uniformly distributed throughout the entire IOL without clear un-opacified areas at the edges, including the optic and the haptic area, in 4 explanted IOLs, based on microscopic evaluation, regardless of calcium staining. These characteristics differed from those of previously reported cases of calcification of IOLs made of the same material as the IOLs in our cases; the opacification may thus be due to a problem in the manufacturing process or in the material itself.

The manufacturers of these lenses have conveyed notices on field safety 3 times to date: in November 2012, December 2014, and September 2017. In the second and third notices, the manufacturers indicated that a plausible interaction between phosphate crystals resulting from the hydration process of the IOLs and batch fluctuations in silicone residues on the IOLs. Given that these processes could expedite precipitation of calcium phosphate, the manufacturers spontaneously withdrew IOLs with a shelf life from January 2017 to May 2020. All IOLs in our report were manufactured during that period, and SEM and EDX confirmed tiny round, calcium deposits similar to those described in previous reports of opacified monofocal IOLs.^[20–22]^ Interestingly, our cases also demonstrated that it may take about 4 years from uneventful initial cataract surgery to the point where subsurface opacification causes significant visual symptoms, which is similar to the findings of a previous report.^[22]^ However, our cases presented a slight difference from the opacification in previous reports; calcification was not limited to the pupillary aperture of the IOL optic, but occurred uniformly throughout the whole IOL, without clear localized areas. The manufacturer's recall of IOLs, including multifocal IOLs, in September 2017 was based on in vitro analysis, and no clinical cases were reported. Since the global market demand and supply of multifocal IOLs have increased markedly since the mid-2010s, it is possible that cases with opacification may continue to increase over the next few years.

There were no significant associations with medical history, medication, and ophthalmic history, such as retinal surgery, Descemet stripping automated endothelial keratoplasty (DSAEK), or Descemet membrane endothelial keratoplasty (DMEK), that could have induced secondary opacification by air or gas. According to previous reports, systemic diseases, such as diabetes, might stimulate the development of opacification. However, in this report, only 2 patients with bilateral significant opacities had diabetes or nonproliferative diabetic retinopathy on slit-lamp examination.

In our cases, we also found alterations using clinical evaluation modalities, such as AS-OCT and OQAS. In 2 previous reports, the authors suggested that using AS-OCT could help to diagnose IOL opacification and prevent misdiagnosis of opacification for posterior cortical opacity.^[23,24]^ We could identify interference by opacification of the IOL surface on retinal OCT images. In affected eyes, macular OCT showed significant signal reduction due to IOL opacification, as compared with the unaffected eye. On the other hand, AS-OCT images showed significant surface hyper-reflectivity by IOL opacification as compared with the clear IOL. In addition, in patients with bilateral IOL opacification confirmed by slit-lamp examination, we found that, on OQAS, the MTF cut-off values and Strehl ratios in the symptomatic eyes were lower than in eyes without symptoms. This diagnostic tool could help to recognize IOL opacification and demonstrate symptoms related to visual quality, such as worsening of contrast sensitivity or visual acuity, which may help to decide whether IOL exchange should be performed.

Multifocal IOL implantation after cataract extraction may be convenient to restore full-range vision, without the need for presbyopia-correcting glasses. However, the Korean National Health Insurance has refused to cover the additional cost of presbyopia-correcting IOLs, as these are 1.5 times more expensive than monofocal IOLs. Consequently, it is a major out-of-pocket expense; thus, the development of postoperative complications after the implantation of multifocal IOLs is a very sensitive issue and the occurrence of IOL opacification raises issues of serious medical litigation. In our cases, 3 of 7 patients filed a lawsuit and were transferred to a tertiary medical institution due to surgical difficulties and collapse of rapport between the doctor and the patient. From a surgical point of view, the LS-313 MF30 has a plate haptic design with substantial thickness and volume. Moreover, the challenge of removing the IOL during IOL exchange several years after initial IOL implantation is further increased due to capsular adhesions. If zonular dehiscence or corneal decompensation occurs, or if the secondary IOL cannot be inserted into the bag due to the Nd:YAG laser capsulotomy or capsular dehiscence, more complicated legal problems can arise.

The limitation of this case series is that a small number of opacification cases were included. It is known that the medical conditions of the patients are associated with the occurrence of IOL opacification. However, because only a small number of cases were included, it is difficult to evaluate precisely related medical conditions. In addition, it is unclear whether the cause of this subsurface calcification is due to phosphate remnants originating from the detergent as shown in the second Field Safety Notice released by Oculentis GmbH in 2017.

In conclusion, we here reported on 7 cases of primary calcification in implanted hydrophilic acrylic refractive multifocal IOLs with a hydrophobic surface. Clinicians must be aware of the potential for opacification of this IOL design, despite hydrophobic surface modification.

## Author contributions

**Conceptualization:** Jong Hwa Jun.

**Investigation:** Seung Pil Bang, Kun Moon, Jong-Ho Lee, Jong Hwa Jun.

**Project administration:** Jong Hwa Jun.

**Resources:** Kun Moon, Jong-Ho Lee.

**Supervision:** Jong Hwa Jun, Choun-Ki Joo.

**Validation:** Seung Pil Bang, Choun-Ki Joo.

**Visualization:** Seung Pil Bang.

**Writing – original draft:** Seung Pil Bang.

**Writing – review & editing:** Jong Hwa Jun, Choun-Ki Joo.

Seung Pil Bang orcid: 0000-0002-7521-544X.
